# PD26 - Acute allergic reactions in children following a cow’s milk exclusion diet for intolerance symptoms such as gastro-oesophageal reflux

**DOI:** 10.1186/2045-7022-4-S1-P26

**Published:** 2014-02-28

**Authors:** Nerys Beynon, Anna Thusby-Pelham

**Affiliations:** 1 Royal Hospital for Sick Children, Bristol, United Kingdom

## 

Clear guidelines have been published by ESPGHAN [1] and RCPCH [2] concerning the diagnostic approach and management of Cow’s milk protein allergy (CMPA) in infants and children. Immune reactions may be IgE mediated, non IgE mediated or mixed and present either with acute allergic reactions or delayed manifesting as eczema “flare ups”, or often non-specific gastrointestinal manifestations such as Gastro-oesophageal Reflux (GORD).

We have observed 5 infants (over 18 months) where there has been emergence of IgE mediated allergic symptoms on reintroduction of cow’s milk following an exclusion diet for intolerance symptoms such as GORD. All infants had been sensitized to cow’s milk, and had been able to tolerate it without immediate hypersensitivity prior to the exclusion diet. Not all had allergy testing prior to challenge.

This has not been described in the literature following exclusion for GORD, but there have been reports of acute allergic reaction following exclusion diet in the management of severe eczema. Flinterman el al. [3] describe a study where children who had been previously sensitized to cow’s milk had this excluded as part of management of Atopic Eczema Dermatitis Syndrome. On re-introduction of CMP often after several years, the children all had acute allergic reactions. This occurred following accidental exposure and during formal food challenge. The children had been able to tolerate CMP without acute reaction prior to exclusion.

This finding has led us to consider changing our practice. We will now perform allergy testing of all children (SPT +/-RAST) who have been following an exclusion diet for intolerance symptoms before proceeding to formal milk challenge. This is because of the chance of developing acute allergic reactions. Parents should be counselled of this risk.
